# Women’s experiences with unexpected induction of labor: A qualitative study

**DOI:** 10.18332/ejm/161481

**Published:** 2023-03-24

**Authors:** Christin Ø. Lundh, Ane-Karine Øvrum, Bente Dahl

**Affiliations:** 1 Centre for Women’s, Family and Child Health, Faculty of Health and Social Sciences, University of South-Eastern Norway, Borre, Norway

**Keywords:** labor, decision-making, induction, information, qualitative, woman-centered

## Abstract

**INTRODUCTION:**

Induction of labor is the most common intervention in modern obstetrics and is a growing phenomenon worldwide. Research on women’s experiences with the induction of labor is scarce, especially on being unexpectedly induced. The purpose of this study is to explore women’s experiences with unexpected induction of labor.

**METHODS:**

We conducted a qualitative study including 11 women who had undergone an unexpected induction of labor within the last three years. Semi-structured interviews were conducted in the period February–March 2022. Data were analyzed using systematic text condensation (STC).

**RESULTS:**

The analysis led to four result categories. The decision to induce labor came as a surprise to the women, both for better and for worse. Information was not automatically provided and was often obtained through the women’s own efforts. Consent to the induction mainly took the form of a decision by healthcare personnel, and the birth was a positive experience during which the woman felt looked after and reassured.

**CONCLUSIONS:**

The women were very surprised when told they had to be induced and were unprepared for the situation. They received insufficient information, and several experienced stress from the time of induction up until they gave birth. Despite this, the women were satisfied with the positive birth experience, and they emphasized the importance of being looked after by empathetic midwives during childbirth.

## INTRODUCTION

Artificial induction of labor is the most common intervention in modern obstetrics and is a growing phenomenon worldwide^1^. The World Health Organization (WHO) estimates that approximately 25% of births in developing countries are induced^2^. The corresponding figure in Norway in January 2022 exceeded this average, at 28.3%^3^. The number continues to grow and has risen by more than 10% over a 10-year period^4^.

Induction of labor must have a clear medical indication and can be performed using a variety of methods^1^. Physiological maturation of the cervix is a normal process that occurs in pregnant women before the onset of labor and one that is stimulated by induction. This often involves mechanical stretching or use of a balloon to release prostaglandins locally. Other methods include applying prostaglandins, amniotomy and oxytocin infusion. The method chosen must be justified according to a scoring system that assesses the degree of maturity^4^. Induction increases the risk of complications during labor, such as uterine hyperstimulation and fetal distress, and is associated with discomfort for the woman due to close monitoring and restricted mobility^2^. Elective induction of labor in healthy pregnant women between 37+0 and 40+6 weeks of gestation is not associated with maternal or neonatal morbidity or an increased risk of cesarean section, but the incidence of admission to a neonatal intensive care unit is higher^5^. Labor has been reported to last longer in women who were induced than in women whose labor started spontaneously^6^.

Research shows that the choice of methods for induction and maternal or fetal outcomes are well documented, but there is less focus on women’s experiences with induced labor^7^. In a Swedish study, women cited induction as a cause of negative birth experiences^8^, but some women do have positive experiences^9^. Negative experiences with labor induction can impact on the woman and her health in the short- and long-term^1^. Studies point to a lack of information and communication, and women do not feel included in the decision-making surrounding the induction process^8,10^. Women are vulnerable during the induction process and need pain relief, information and predictability^10^. However, maternity care has undergone a paradigm shift in which technology is playing a larger role and care is being standardized^11^. The purpose of this study is to explore women’s experiences with the unexpected induction of labor.

## METHODS

### Study design

The qualitative method was used to shed light on the research problem. The aim was to explore the diversity and nuances of women’s subjective experiences of unexpected induction^12^.

### Setting, recruitment and population

We used convenience sampling to recruit respondents from Facebook and Instagram. A call for participants was published along with contact details on our personal accounts and added to the Facebook group ‘Graviditet, fødsel og tiden etter’ (Pregnancy, birth and the postnatal period) with a request to share the post. Thirty-three women responded, either to participate in the study or to request more information. Thirteen women met the inclusion criteria which were: 1) induction had taken place within the last three years; 2) pregnant with a live fetus born after gestational week 37; 3) induction and labor took place in a hospital; and 4) healthy during pregnancy. One woman withdrew from the study before the interview due to a traumatic birth experience. The sample consisted of five women giving birth for the first time and six women giving birth for the second time. The women lived in Southern, Eastern, Western and Northern Norway, and had given birth both in large and small hospitals. The births had taken place between three months and three years ago. All the women were aged <40 years and none were defined as high risk.

### Data collection

We conducted individual semi-structured interviews in the period February–March 2022 via Zoom. Two of the authors were present. Zoom interviews were preferred due to the pandemic situation, and also because it enabled the inclusion of women from a wide geographical spread in Norway. The interviews lasted between 17 and 41 minutes (average 29). An interview guide was used, and the interviews began with an open-ended question: ‘Can you tell us about your experience of having your labor induced unexpectedly? We would like you to describe the process to us, focusing on your perceptions of the situation from when you were told that you would be induced up until you gave birth’. We started with this question in order to obtain the women’s rich and spontaneous descriptions of their experiences^13^. Follow-up questions were used for elaboration and clarification. Audio recordings were made of the interviews, and these were transcribed after each interview.

### Data analysis

The interviews were analyzed using systematic text condensation (STC)^14^, which is a four-step thematic cross-case analysis method. In the first step, an overall impression was gained by reading the interview transcripts, and preliminary themes were identified: information, shock, care, relationship, and autonomy. In the second step, meaning units shedding light on the research problem were identified and organized in code groups. We discussed this thoroughly before reaching agreement on the code groups. In the third step, the meaning units in each code group were sorted into subgroups. This process was carried out jointly and in several rounds. The meaning units in each subgroup were then synthesized into a condensate, an artificial quotation using the participants’ words. In the fourth step, an analytical text was formulated based on the condensates, and quotes were selected that reflected the content^14^. An overview of code groups and subgroups is presented in Table 1.

**Table 1:** Overview of code groups and subgroups (11 Norwegian women’s experiences with unexpected induction in 2022)

*Code group*	*Subgroup*
**The information:** not given automatically and often the result of own efforts	Information was lacking Using Google helped meet the need for knowledge and reassurance Good information helps the women feel they are being looked after
**Labor:** looked after and reassured when the pain takes over	Feeling looked after but also being given enough space Control and calm atmosphere create reassurance Satisfied despite challenges
**The consent:** mainly a decision made by healthcare personnel	My choice? Information, but lack of informed consent Sense of being on the sidelines
**The decision:** surprising, for better or worse	My birth plan was ruined An unexpected event, but also an opportunity The circular dance of ambivalence

### Ethical approval and informed consent

The study was conducted in accordance with the Declaration of Helsinki^15^. The Regional Committees for Medical and Health Research Ethics considered the study to be outside the scope of the Norwegian Health Research Act (REK: 409556). The Norwegian Centre for Research Data (NSD: 986577) approved the project. The women were sent an information letter and consent form to read prior to the interviews. They were asked to confirm their consent to participate in the project by clicking on the Zoom link sent by email. Information was provided about the option to withdraw from the study without repercussions up until the start of the data analysis.

## RESULTS

The analysis led to four result categories.

1. The decision: surprising, for better or worse;
2. The information: not given automatically and often the result of own efforts;
3. The consent: mainly a decision made by healthcare personnel; and
4. Labor: looked after and reassured when the pain takes over.

### The decision: surprising, for better or worse

The women explained that when they were told that labor would have to be induced it was very unexpected. Some perceived it as dramatic, while others felt uncertainty, disappointment or confusion. Some were caught off guard, while others were upset because it wrecked their wish for a home birth. They did not have time to reflect on the information, and said they were unprepared, both mentally and in practical terms. Several of the women had not brought a hospital bag with them and some had come without their partner because they were only expecting a check-up. Some of the women described an incongruence in relation to the induction and felt that their bodies were not ready for childbirth. One woman said that forcing her body to go into labor seemed brutal:

*‘When she comes in and says “you’re going to have your baby now”, well … ehhh, my world kind of turned upside down because I wasn’t prepared at that point. Going from leaving home with “remember your phone charger in case you need to stay overnight”, to sitting with a baby in my arms in the evening, it was really … It takes you by surprise.’* (Participant 3)

Some of the women liked the thought of not having to carry the pregnancy to term or risk going past their due date. Others felt relief knowing when the birth would take place. One woman had noticed before she came to the check-up that something was not quite right and felt that she was taken seriously when she informed the healthcare personnel of this. She was surprised, but in a good way, and felt that the healthcare personnel would take care of her and her baby. Some women felt reassured by the prospect of an induced labor as it meant they could stay in hospital to be monitored and avoid the risk of giving birth outside the hospital setting:

*‘Because my due date was on a Monday, I was allowed to wait until the blood samples had at least been analyzed. Then they called me on Saturday night and said that I would be getting induced on Sunday morning. So I was aware that I would likely be induced. We actually thought it was fine, because I have a child at home as well.’* (Participant 9)

### The information: not given automatically and often the result of own efforts

Most women felt they were given insufficient information, particularly in relation to the ongoing process and risk factors. Some received an information leaflet about the use of a balloon but did not feel that this replaced the need for oral information. One woman got a shock when the balloon was being removed. She had heard the word ‘balloon’ but did not understand that it was actually a balloon; no one had shown her what it looked like. Few women had received information about the increased risk of cesarean section or other risk factors linked to induction. Very few were allowed to have a partner present during the consultations, which they explained was due to the COVID-19 pandemic. One woman discovered in conversations with doctors and midwives that her situation was serious, but when her induction was postponed several times, it was difficult to know what to think. The women put the lack of information down to the heavy workload in the hospital department, they did not want to be a bother:

*‘They just kind of assumed that when they said they’d start me off when they had time that I would understand what that entails.’* (Participant 11)

The women said that they used Google to find the information they needed. They googled different induction methods, women’s experiences of induced labor and risk factors for induction. One woman was so unsure about why she was being induced that she tried to google what was actually wrong with her:

*‘When I think about it, I didn’t feel reassured until I went home and googled it.’* (Participant 5)

Few women felt that they received sufficient information during their labor. One woman had a need for a conversation about specifics, which she referred to as an induction conversation. She felt that such a conversation should include general information about what induction is and what can be expected. One woman received good information about the process before going home with a balloon, another felt well supported after she was induced because she felt that she was now ‘in the system’. Once labor was underway, the women felt well informed and supported. Pain and contractions were cited as possible reasons why they were not given information as their labor progressed. One woman experienced being so deep into her ‘labor bubble’ that she was not receptive to information. Another said that the stress of the situation made her forget everything that the healthcare personnel told her, but she described how the midwife took the time to repeat the information:

*‘So I asked her again because when I was told, all the information I had been given disappeared, hehe, inside my head. So she explained everything again. She was really, really good.’* (Participant 7)

### The consent: mainly a decision made by healthcare personnel

Most women underwent a clinical assessment in connection with possible induction and felt part of the decision-making. This helped give them an overview of what was going on and made them feel reassured. During the induction process, some found that procedures were carried out that they had not consented to or had not had time to think about beforehand, but they considered it a necessary part of the process. The women said that they received insufficient information to make a fully informed choice and give their consent, but some felt that choosing not to be induced would put the child at risk. They experienced that the information they were given about induction was presented as a decision that had already been taken by healthcare personnel and felt that they had no real opportunity to influence the decision-making:

*‘… No, so it was, in a way … it was … it was communicated as a decision. Or such like … yes, without any drama, but there were no questions or anything, I never got the impression that I could influence it in any way … So it was a message that was relayed, in a way.’* (Participant 10)

The women associated the decision to induce them with reassurance and had confidence in the clinical recommendations. They had confidence in the healthcare personnel and trusted that they knew what was best. However, some described being treated with ambivalence or not being taken seriously. They were unsure whether induction was the right choice and wondered whether everything could still go well. The vaginal examination was a procedure they had no control over. It was painful, and being able to catch their breath and mentally comprehend what was happening was a challenge. One woman described how things were done to her body that she did not understand. This made her feel that she was losing sight of herself and her own body:

*‘Because I felt that I didn’t get … I mean, I wasn’t fully aware … I didn’t have time to prepare myself mentally for what was going to happen, before it felt like I had a whole arm up there. You know, and it was physically painful, it felt as if someone was doing whatever they wanted with my body in a horribly vulnerable situation. I lost both that (autonomy) and myself and my body and everything …’* (Participant 2)

### Labor: looked after and reassured when the pain takes over

The women described how they were looked after by the midwives during labor. Being looked after by someone who understood that the women needed help and care by listening to them and recognizing when they were scared was invaluable. The right balance between feeling looked after and being given enough space during labor was crucial. They felt that the midwives understood when they needed care, and when the chemistry was right between the woman and the midwife, the birth was an unforgettable experience:

*‘I actually miss her; she was a really lovely midwife. She was calm, told me what was going to happen. What she thought … And yes, was exactly what you needed, encouraging and supportive. Truly an angel.’* (Participant 5)

It was important to the woman and her partner that healthcare personnel gave the impression of being in control, especially during labor. One woman appreciated having a midwife present throughout active labor, while another woman found it reassuring that she had met the midwife previously. The women described it as meaningful that the midwives met their needs during labor and found it reassuring to be well looked after by experienced midwives and doctors with a calm demeanor. One of the women described the doctor’s presence as follows:

*‘Then I remember that the doctor was there, but he was standing with his hands crossed. So it, it kind of created a calm atmosphere. He didn’t stress my husband out. My husband was also very reassured by him just standing there watching. But he was there in case she was small or something else happened.’* (Participant 3)

Several women experienced pain and overstimulation during labor, and one woman said that she was overwhelmed by the fact that there was no gap between contractions as she had expected. A midwife told one of the women to be prepared for an arduous labor. She described the oxytocin infusion as a train that starts moving and does not stop until the baby is born. Another woman found that her labor happened so fast that she was unable to do anything other than breathe, despite being warned about the intensity of the contractions at the time of induction. The birthing process was an intense experience for everyone, and several women ended up undergoing assisted vaginal birth or cesarean section. Still, all were satisfied with their birth experience:

*‘I was, I’ve been incredibly lucky, that the induction went so well and my body just did its job even though, even though I had been induced.’* (Participant 4)

## DISCUSSION

The women in this study reported that induction was recommended based on a clinical assessment of the health of the mother or the baby. However, they felt that they were given insufficient information about the induction, including details of the reason, method, process, risk factors, and potential complications. Earlier research has demonstrated that labor induction is an intervention with the potential to negatively impact on women’s birth experience^16^, and insufficient information and knowledge about the various aspects of induction were cited as reasons for negative experiences^7^. A study demonstrated that women rarely read information leaflets distributed on maternity wards prior to induction^17^. A small number of the women in our study were given an information leaflet, but they still would have liked to receive oral information. They felt that the staff were rushed, they were afraid of being a bother and they believed that the heavy workload on the maternity wards was part of the reason why they did not receive sufficient information. Midwives describe how they have to deprioritize tasks because they have so much to do^18^. Handing out information leaflets rather than giving oral information can therefore save time^17^. In an attempt to obtain more information, the women in this study said that they used Google. However, the problem with googling is that not all information comes from reliable sources, and it can be difficult to link the information to a person’s specific situation^17^.

Some women felt that the clinical recommendation for induction was formulated as a decision that had already been made, and the focus was on the risk to the child if induction did not take place. Some felt that they were unable to influence the decision, while others felt that they participated in the process. This is consistent with earlier research that highlights how women want to participate in the decision-making process^8^. It is also consistent with research on women’s experiences of making informed choices, which shows that sufficient and individualized information given at the right time promotes predictability and participation^19^. It emerged in the interviews that some women did not remember whether they had received information or been asked for consent. They linked this to the time at which information was given and if they simultaneously experienced pain etc. This shows that the timing, quality and quantity of information provided is important^19^. The women in the study said that clinical assessments were made prior to induction. Nevertheless, they were ‘caught off guard’ when told of the recommendation to induce them. They felt they did not have enough knowledge to consider alternatives to induction and were unable to make an informed choice. However, they felt reassured and had confidence in the health service, the recommendation and the treatment they received, although some felt they were treated with ambivalence in relation to the decision to induce them. For some, induction was a high-risk event that made them reflect on possible adverse incidents; the process was, after all, a way of forcing something that they felt their body was not ready for. Others felt a sense of relief at being induced, and induction was therefore a motivating factor in the birthing process.

Information is a key component in promoting woman-centered care^20^. In a health service that has undergone a paradigm shift with an increasing focus on medicalization, including in maternity care^11^, midwives play an important role in promoting women’s opportunities for participation and ability to make informed choices. When midwives took the time to recognize the needs of the women in this study, this gave the women a sense of reassurance and predictability, which had a positive impact on their experience. In the study by Moore et. al.^21^, the authors conclude that the choices women make are often influenced by the medical advice they receive. The women felt that they had no choice, and that induction was unavoidable due to the focus on risk^21^. This is consistent with our findings, which show that the influence of healthcare personnel and their perception of risk have an impact on women’s choices and decision-making^19^. The women in our study had a general idea of why they should be induced, and several opted for this because it was recommended to them by healthcare personnel. The results show that the woman’s ability to understand the information and knowledge provided was partly dependent on her particular situation. In line with the main principles of woman-centered care, individualization of care is crucial. To counteract the threat of standardized maternity care^11^, midwives can adopt a holistic approach and identify each woman’s needs in her particular situation.

For the women in the study, the care provided by the midwife was an important part of their birth experience. Only a small number of the women had met their midwife before, but all felt supported and well looked after. A good and reassuring relationship with the midwife can be established quickly, with the foundation for the relationship being laid when the midwife introduces herself^22^. Confidence in the midwife can be crucial for the women’s birth experience and for establishing good relations^23^. It was important that the midwife ensured the right balance of providing care and giving the woman enough space during labor. The midwife’s ability to recognize when women needed care, her clarity and the good relationship between the two were considered important by the women. Midwives should facilitate a reassuring and relaxing atmosphere in order to promote the release of oxytocin. Pain and uncertainty can inhibit the release of oxytocin and prolong labor^17^. If the midwife has faith in the woman, this strengthens the woman’s faith in herself during childbirth^22,24^. A busy ward will not necessarily impact on the woman and her birthing partner if the midwife establishes a calm atmosphere in the delivery room and gives them her full attention. The quality of the midwife’s presence may be more important than the quantity^25^.

Women’s birth experiences are a key component in quality-of-care assessments in maternity departments^1^. Cervical maturation is associated with less positive induction experiences, but not because of the interventions themselves^1^. This is also reflected in the results of this study. The women faced challenges during the induction process, but they were nevertheless satisfied with their birth experience. The women who underwent surgery explained that their satisfaction stemmed from the fact that they had been well looked after by healthcare personnel during labor and that they had had the opportunity to experience labor before surgery. The women who had quick deliveries were pleased that their body just did its job despite being induced. Almost all of the women in the study were satisfied with their birth experience, despite undergoing surgery, assisted vaginal births or painful procedures, and receiving insufficient information. Coates et al.^7^ claim that giving birth to a healthy child can be enough for a woman to feel satisfied with the birth experience. In our study, the women also emphasized how the midwife’s presence and care contributed ultimately to their having a positive birth experience.

### Strengths and limitations

A qualitative design and individual interviews were suitable for exploring women’s experiences with unexpected induction. The interviews were conducted via Zoom, which probably made it more difficult to interpret non-verbal language due to the barrier represented by non-physical meetings. However, conducting the interviews remotely enabled us to include women from a wide geographical spread, and we found that the interviews provided rich data for shedding light on the research problem. The women had variation in parity and were induced for different reasons. They also lived in different parts of the country and had given birth in different hospitals. However, all the participants were ethnic Norwegian, and it would have strengthened the study if women of different ethnicities had been included.

## CONCLUSIONS

Women who are induced unexpectedly find themselves in a situation they were not prepared for. They lacked knowledge of the procedure, and the information they received from healthcare personnel was not adequate for them to make informed choices in those circumstances. The implementation of a pre-induction conversation based on shared-decision making may be a good alternative for this group of women. The conversation should include sufficient and individualized information about the procedure as well as an explanation of why induction is recommended. Despite the lack of knowledge and information, the women felt reassured and well looked after by the midwives. They trusted healthcare personnel’s knowledge and had confidence in clinical recommendations. By promoting women’s opportunities for active participation in healthcare issues and taking the time needed to recognize the woman’s needs, the midwives are working in compliance with the requirements for professional standards and best practice in woman-centered midwifery care. This can help ensure that women experience reassurance and predictability even in challenging situations.

## Data Availability

Data cannot be made available due to privacy reasons.
